# Adverse Effects After Prehospital Administration of Naloxone by Bystanders: A Preliminary Study

**DOI:** 10.1017/S1049023X24000128

**Published:** 2024-04

**Authors:** Daniel Du Pont, Rebecca Fenderson, Krystal Hunter, Alexander Kuc, Gerard Carroll

**Affiliations:** 1. Department of Emergency Medicine, University of Pennsylvania, Philadelphia, Pennsylvania USA; 2. Department of Emergency Medicine, Cooper University Hospital, Camden, New Jersey USA; 3. Cooper Research Institute, Cooper Medical School of Rowan University, Camden, New Jersey USA; 4. Division of EMS and Disaster Medicine, Department of Emergency Medicine, Cooper Medical School of Rowan University, Camden, New Jersey USA

**Keywords:** bystander, naloxone, opiate, opioid, overdose

## Abstract

**Objective:**

Opioid use disorder is a cause of significant morbidity and mortality. In order to reverse opioid overdose as quickly as possible, many institutions and municipalities have encouraged people with no professional medical training to carry and administer naloxone. This study sought to provide preliminary data for research into the rates of adverse effects of naloxone when administered by bystanders compared to Emergency Medical Services (EMS) personnel, since this question has not been studied previously.

**Methods:**

This was a retrospective cohort study performed at an urban, tertiary, academic medical center that operates its own EMS service. A consecutive sample of patients presenting to EMS with opioid overdose requiring naloxone was separated into two groups based on whether naloxone was administered by bystanders or by EMS personnel. Each group was analyzed to determine the incidence of four pre-specified adverse events.

**Results:**

There was no significant difference in the rate of adverse events between the bystander (19%) and EMS (16%) groups (OR = 1.23; 95% CI, 0.63 - 2.32; P = .499) in this small sample. Based on these initial results, a study would need a sample size of 6,188 in order to reach this conclusion with 80% power. Similarly, there were no significant differences in the rates of any of the individual adverse events. Secondary analysis of patients’ demographics showed differences between the two groups which generate hypotheses for further investigation of disparities in naloxone administration.

**Conclusions:**

This preliminary study provides foundational data for further investigation of naloxone administration by bystanders. Adverse events after the prehospital administration of naloxone are rare, and future studies will require large sample sizes. These preliminary data did not demonstrate a statistically significant difference in adverse event rates when comparing naloxone administration by bystanders and EMS clinicians. This study provides data that will be useful for conducting further research on multiple facets of this topic.

## Introduction

The opioid use disorder crisis continues to drive significant morbidity and mortality in the United States. Unintentional injury is the leading cause of death for people between the ages of one and 44,^1^ with overdoses causing the majority of those deaths.^2^ Emergency Medical Services (EMS) patient encounters for opioid overdose are correspondingly common, and these patients’ one-year mortality has been found to be close to 10%.^3^

Naloxone, the antidote for opioid overdose, was initially only available as a treatment administered by medical personnel. Thanks to public health efforts, non-medical first responders and lay persons routinely carry and administer naloxone. These efforts recently culminated in the United States Food and Drug Administration’s (FDA; Silver Spring, Maryland USA) approval of the first over-the-counter naloxone nasal spray.^4^ This approval was based on an overall risk-benefit assessment of allowing lay persons to administer naloxone, including the clear danger that opioid use presents to public health.

As part of the application for the switch from prescription to over-the-counter, the manufacturer (Emergent BioSolutions; Gaithersburg, Maryland USA) provided data supporting the safety of naloxone. These data included a human factors study of the labeling and instructions with 71 participants as well as post-marketing safety monitoring. The post-marketing data likely under-estimate the incidence of adverse events by a significant margin. For example, the manufacturer’s safety database notes 27 cases of serious opioid withdrawal after naloxone administration out of approximately 27 million doses.^5^ Common clinical experience shows that the rate of severe withdrawal after naloxone administration is much more than one in one million.

While many studies have examined the efficacy and safety of naloxone administration by health care professionals, the literature concerning adverse effects of bystander-administered naloxone is extremely limited. There exist only a few small observational studies of the degree of withdrawal symptoms in patients who received bystander naloxone.^6–8^ The objective of this preliminary study was to examine the rates of adverse events when naloxone was administered by bystanders compared to prehospital clinicians in order to provide baseline data for further research.

## Methods

### Study Design and Setting

This was a retrospective cohort study performed at an urban, tertiary, academic medical center (Cooper University Hospital) that runs a hospital-based EMS service (Cooper EMS) serving as the sole 9-1-1 response EMS agency for the city of Camden, New Jersey (USA). Cooper EMS is a two-tiered service primarily utilizing emergency medical technicians and paramedics with additional support from a 24-hour paramedic supervisor response vehicle and EMS physician response units. From 2020 through 2022, Cooper EMS responded to an average of 1,211 overdose calls per year. The population of Camden over this time period was approximately 71,000.^9^ The Cooper University Hospital Institutional Review Board approved this research study (#22-227).

### Selection of Participants

Patient encounters were identified by searching Cooper EMS records (emsCharts; Zoll Medical; Chelmsford, Massachusetts USA) for calls with a primary or secondary clinical impression of drug overdose, poisoning by heroin, or substance abuse. Due to the lack of prior studies of this topic, a precise power calculation was not possible. Based on prior studies of naloxone administration by trained medical personnel, it was decided to perform a preliminary study of 100 patients that received naloxone only from bystanders.

Patients were included if they were given naloxone by one of either bystanders (bystander group) or EMS clinicians (EMS group). In Camden, police officers carry and administer naloxone. For the purposes of this study, they were classified as bystanders. Patients were excluded if they were prisoners, known to be pregnant, under the age of 18, in cardiac arrest on EMS arrival, or if they had received naloxone from both a bystander and an EMS clinician. Since Cooper EMS is a two-tiered service, some patient encounters had two charts (one each for the responding Basic Life Support [BLS] and Advanced Life Support [ALS] units). If conflicting information was found in two charts from a single encounter, it was excluded.

### Data Collection

Data were abstracted using standardized forms. Objective data including call times, patient demographics, vital signs, naloxone dosing, and patient disposition were abstracted by one emergency medicine trained EMS fellow who was aware of the purpose of the study. As part of their standard documenting practice, EMS clinicians specified in the medical record who administered each dose of naloxone. Initial vital signs were only included if they were taken within five minutes of EMS arrival. When incomplete information was found in the EMS chart, hospital records were searched using an online record-sharing system that includes all three of the EMS receiving centers in Camden. Demographic data could be retrieved for patients who refused transport as long as the EMS crew was able to obtain name and date of birth and the patient had previous contact with Cooper EMS or with any of the receiving hospitals in Camden.

Presence or absence of adverse effects using standardized definitions was abstracted on a separate form by one emergency medicine resident who was blinded to the purpose of the study. Charts were reviewed for four pre-specified adverse events: nausea/vomiting, seizure, agitation/aggression, and pulmonary edema (Table 1 includes definitions). The definitions were chosen to be consistent with, but more specific than, those in prior studies of adverse events after naloxone administration by EMS clinicians.^10–14^

**Table 1.** Study Definitions

● Nausea/Vomiting: Any positive mention of either
● Seizure: Any abnormal movements defined as seizure or seizure-like
● Agitation/Aggression: Documentation of any one of actual or attempted violence by the patient, the need for chemical or physical restraint, agitation significant enough to impact clinical care, or any other evidence of clinically significant agitation
● Pulmonary Edema: The onset of respiratory failure requiring noninvasive positive pressure ventilation or intubation that did not have a clear alternative cause

Note: All of the above are only valid if documented as happening after administration of naloxone.

An emergency medicine trained EMS faculty member reviewed a randomly selected 20% of charts for accurate data abstraction. He was aware of the purpose of the study; however, any information about naloxone administration was redacted from charts before he viewed them. All three abstractors and reviewers received training on emsCharts, study definitions, and abstraction forms prior to data collection.

### Data Analysis

Data were analyzed using SPSS 27 (IBM Corp.; Armonk, New York USA) and summarized with frequencies and percentages. For continuous data, mean and standard deviation (SD) are presented. An independent t-test was used to analyze differences. For categorical data, a chi square test was used.

## Results

### Characteristics of Study Subjects and Naloxone Dosing

**Figure 1.**
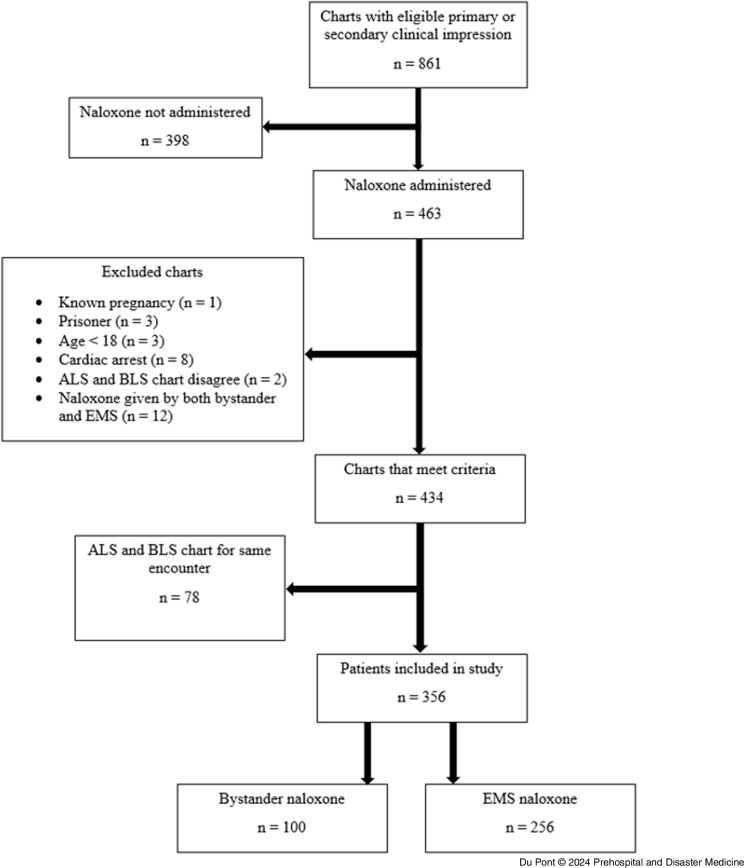
Patient Selection. Abbreviations: ALS, Advanced Life Support; BLS, Basic Life Support; EMS, Emergency Medical Services.

In order to reach the pre-specified number of patients who received naloxone from bystanders only, EMS charts were consecutively sampled from January 1, 2022 through April 28, 2022. From the 861 charts in this range with an appropriate clinical impression, 356 unique patient encounters that met all study criteria were derived (Figure 1). There were 100 patients in the bystander group and 256 in the EMS group.

Study patients’ demographics are shown in Table 2. Male patients were much more common than female patients in this study, with males making up 73.0% of the bystander group and 80.1% of the EMS group.

**Table 2.** Patient Characteristics

	Bystander Group			EMS Group			
	N	n	Percent	N	n	Percent	P Value
Sex	100			256			
Male		73	73.0		205	80.1	.147
Female		27	27.0		51	19.9	
Race/Ethnicity	95			248			
Black		34	35.8		123	49.6	.022
White		42	44.2		70	28.2	.005
Hispanic		18	18.9		55	22.2	.513
Asian		1	1.1		0	0.0	.277

Abbreviation: EMS, Emergency Medical Services.

The distribution of Black and White patients was also heavily skewed between the groups. Patient race was documented in 95% (95/100) of the bystander group and 97% (248/256) of the EMS group. Of these, 49.6% of the EMS group was Black, compared with 35.8% of the bystander group (P = .022). Conversely, 44.2% of the bystander group was White, compared with 28.2% of the EMS group (P = .005).

The number of naloxone doses and total naloxone dose given to each patient are detailed in Table 3 and Table 4, respectively. All but two patients in the bystander group received one (73%) or two (25%) doses of naloxone. The most common total doses were 4mg (67%) and 8mg (24%). In the EMS group, most patients received one dose (94.5%) with the most common total dose being 2mg (85.9%).

**Table 3.** Number of Naloxone Doses

	Bystander Group			EMS Group		
	N	n	Percent	N	n	Percent
Doses	100			256		
1		73	73.0		242	94.5
2		25	25.0		11	4.3
3		1	1.0		2	0.8
4		0	0.0		1	0.4
5		1	1.0		0	0.0

Abbreviation: EMS, Emergency Medical Services.

**Table 4.** Total Naloxone Dose

	Bystander Group			EMS Group		
	N	n	Percent	N	n	Percent
Total Dose	100			256		
0.4		0	0.0		4	1.6
0.5		0	0.0		5	2.0
1.0		0	0.0		11	4.3
2.0		5	5.0		220	85.9
2.4		0	0.0		1	0.4
2.5		0	0.0		1	0.4
3.0		0	0.0		5	2.0
4.0		67	67.0		8	3.1
5.0		0	0.0		1	0.4
8.0		24	24.0		0	0.0
12.0		1	1.0		0	0.0
Unknown		3	3.0		0	0.0

Abbreviation: EMS, Emergency Medical Services.

### Primary Results

The purpose of this study was to provide preliminary data for research into the rates of adverse effects of naloxone when administered by bystanders compared to EMS personnel, since this question had not been rigorously studied before. The primary outcome being examined in this study was the rate of adverse events after naloxone administration.

There was no significant difference between the bystander and EMS groups, with adverse events occurring in 19% of the former and 16% of the latter (OR = 1.23; 95% CI, 0.63 - 2.32; P = .499). Based on the results from this initial cohort of 356 patients, a sample size of 6,188 patients would be required to reach this conclusion of 80% power; this large number was not surprising given the small effect size that was found. This preliminary study was designed to enable power calculations for further studies; all other outcomes and calculations should be viewed as hypothesis-generating only.

There were no significant differences in the rates of any of the individual adverse events between the EMS and bystander groups (Table 5) or between the overall rate of adverse events in patients who received 4mg or less of naloxone compared to those who received more (Table 6). No patients developed pulmonary edema by this study definition. One patient in each group required intubation in the field; however, both were found down with vomit in their mouth before naloxone was administered, were intubated in the field, and had CT scans in the emergency room that were consistent with significant aspiration.

**Table 5.** Patient Outcomes

	Bystander Group			EMS Group						
	N	n	Percent	N	n	Percent	P Value	Odds Ratio	95% CI	
Adverse Events	100			256						
Any Event		19	19.0		41	16.0	.499	1.23	0.63	2.23
Nausea/Vomiting		10	10.0		20	7.8	.504			
Seizure		1	1.0		1	0.4	.483			
Agitation		11	11.0		22	8.6	.482			
Pulmonary Edema		0	0.0		0	0.0				
Disposition	100			256						
Refusal		20	20.0		79	30.9	.040			
Refusal by Action		13	13.0		19	7.4	.098			
Transport by ALS		29	29.0		120	46.9	.002			
Transport by BLS		38	38.0		38	14.8	<.001			

Abbreviations: ALS, Advanced Life Support; BLS, Basic Life Support; EMS, Emergency Medical Services.

**Table 6.** Adverse Events by Total Naloxone Dose

Total Naloxone Dose	Any Adverse Event	No Adverse Events
≤ 4mg	12	59
> 4mg	7	19

Note: P = .271 (Chi Square Test).

Cardiac arrest, if caused by naloxone administration, would obviously be an important side effect. Patient were excluded if they were found to be in cardiac arrest on EMS arrival because it is very difficult for bystanders to reliably determine the presence or absence of a pulse in a patient in respiratory arrest; it would, therefore, be very difficult to tell whether a patient went into cardiac arrest before or after naloxone administration. On chart review of the eight patients excluded for cardiac arrest, none had details in the History of Present Illness/HPI suggesting that the patient had a witnessed cardiac arrest. No patients in the study went into cardiac arrest after EMS arrival.

Twenty percent (n = 74) of included encounters were audited by an EMS faculty member who was blinded to group allocation. With four types of adverse event possible chart, a total of 296 outcomes were audited; of these, there was disagreement on only one instance of nausea/vomiting (0.3% of total events). A patient in the EMS group was documented as feeling “sick” after naloxone administration, with a final adjudication that this did not meet the study definition.

### Secondary Results

Patients’ vitals and Glasgow Coma Score (GCS) supported the effectiveness of bystander-administered naloxone in reversing respiratory depression and decreased mental status. Compared to the EMS group, patients in the bystander group had higher initial respiratory rates (mean 12.3 versus 6.7; P < .001), oxygen saturations (90.0 versus 78.2; P < .001), and GCS (8.62 versus 4.71; P < .001).

There were also significant differences in patient disposition. Patients in the bystander group were modestly less likely to refuse transport than those in the EMS group (20.0% versus 30.9%; P = .04), but this was somewhat offset by an opposite trend in patients walking away from EMS without completing a formal refusal, also known as refusal by action (13.0% versus 7.4%; P = .098). There were larger differences in the level of transport; the bystander group was transported by ALS much less often (29.0% versus 46.9%; P = .002) and by BLS much more often (38.0% versus 14.8%; P < .001).

Finally, the encounter time differed between the two groups. Since EMS offload times can vary significantly, encounter time was defined as the time between EMS arrival on scene and EMS clearing the scene (for patients who refused) or arriving at the hospital (for patients who were transported). The mean encounter time was 16.5 minutes in the bystander group compared to 21.8 minutes in the EMS group (P < .001).

## Discussion

To the best of the authors’ knowledge, this is the only study of its kind examining the adverse effects of bystander-administered naloxone. A literature search conducted by the authors with the help of a research librarian revealed only three studies related to the topic, all of which looked at incidence of withdrawal symptoms. Abdelal, et al used a web-based survey of 125 adults in the United States who administered naloxone in the year preceding the survey.^6^ Moustaqim-Barrette, et al used forms that could be voluntarily filled out and returned by people who administered naloxone in British Columbia.^7^ The only adverse event reported on by these two studies was withdrawal symptoms. Neale, et al used qualitative data from semi-structured interviews of bystanders who had administered naloxone. Fifteen of 62 cases were excluded because data relating to the patients’ response to naloxone were missing. They reported on the incidence of withdrawal as well as “anger,” which was not otherwise defined.^8^

As discussed in the introduction, naloxone was approved as an over-the-counter product despite extremely limited data regarding potential adverse events when administered by bystanders without medical training. Considering its excellent safety profile when used by trained clinicians and the extreme and common harms resulting from opioid overdoses, this approval was reasonable. That being said, the incidence and severity of adverse events of bystander-administered naloxone must still be studied in order to inform how it can be deployed in the safest way possible. This study adds to this nascent literature by providing the first data collected by trained clinicians using rigorous methods in a consecutive sample of EMS records.

In this preliminary study, there was no statistically significant difference in the number of adverse events experienced by patients who were given naloxone by bystanders compared to EMS clinicians. Studies with significantly larger sample sizes will be needed to confirm this finding with sufficient power. The secondary outcomes also generate hypotheses that require further study. In this sample, White patients were more likely to be administered naloxone by bystanders than Black patients. While disheartening, this is consistent with prior research showing that Black patients were less likely than White patients to receive bystander cardiopulmonary resuscitation in witnessed out-of-hospital cardiac arrest,^15^ and that among patients who were prescribed opioids, Black patients were less likely to be simultaneously prescribed naloxone.^16^

The differences between groups in encounter times and ALS utilization are also notable. The EMS units (particularly ALS units) are a scarce resource in many systems. It would be a notable operational benefit if bystander naloxone administration is found to truly lead to a significant decrease in EMS encounter times and the need for transport by an ALS unit.

## Limitations

The primary limitation of this study is that it is a retrospective chart study. While the clinical equipoise required to study bystander-administered naloxone in a randomized fashion does not exist, collecting the data in a prospective fashion after training EMS clinicians to look for and record adverse events in a standardized fashion would be preferable.

Some patients who received naloxone for presumed overdose during the study period in Camden would not have been captured by reviewing Cooper EMS records. It is unknown how many patients were given bystander naloxone without 9-1-1 being called. Given that EMS was not called, this group likely had a lower rate of adverse events than the bystander group in this study. This study also did not capture EMS patients who were cared for by mutual aid agencies and had no contact with Cooper EMS.

Patient race and ethnicity were recorded by EMS clinicians. In the vast majority of cases, this was done based on the clinician’s impression of the patient’s race and ethnicity as opposed to asking the patient.

Additionally, the high rates of opioid use and overdose in Camden may limit the external validity of these results when applied to areas where both bystanders and EMS clinicians have less experience using naloxone.

## Conclusion

This study provides data that will be useful in planning further research into bystander-administered naloxone. These preliminary data did not demonstrate a statistically significant difference in adverse event rates when comparing naloxone administration by bystanders and EMS clinicians but did generate numerous hypotheses for future study. Larger studies are needed to confirm these findings.
